# Tomographic Task-Related Functional Near-Infrared Spectroscopy in Acute Sport-Related Concussion: An Observational Case Study

**DOI:** 10.3390/ijms21176273

**Published:** 2020-08-29

**Authors:** Mario Forcione, Antonio Maria Chiarelli, David Perpetuini, David James Davies, Patrick O’Halloran, David Hacker, Arcangelo Merla, Antonio Belli

**Affiliations:** 1 National Institute for Health Research Surgical Reconstruction and Microbiology Research Centre (NIHR-SRMRC), University Hospitals Birmingham NHS Foundation Trust, Mindelsohn Way, Birmingham B15 2TH, UK; daviesdj@doctors.org.uk (D.J.D.); a.belli@bham.ac.uk (A.B.); 2 Neuroscience & Ophthalmology Research Group, Institute of Inflammation & Ageing, College of Medical and Dental Sciences, University of Birmingham, Edgbaston, Birmingham B15 2TT, UK; patrickohalloran@doctors.org.uk; 3 Imaging and Clinical Sciences, Department of Neuroscience, University G. D’Annunzio of Chieti-Pescara, Institute for Advanced Biomedical Technologies, Via Luigi Polacchi 13, 66100 Chieti, Italy; antonio.chiarelli@unich.it (A.M.C.); david.perpetuini@unich.it (D.P.); arcangelo.merla@unich.it (A.M.); 4 Clinical Neuropsychology, University Hospitals Birmingham NHS Foundation Trust, Mindelsohn Way, Birmingham B15 2TH, UK; david.hacker@uhb.nhs.uk

**Keywords:** fNIRS, diffuse optical tomography, DOT, traumatic brain injury, TBI, Silent Word Generation, Symbol Search, Digit Span, WAIS-IV, return-to-play

## Abstract

Making decisions regarding return-to-play after sport-related concussion (SRC) based on resolution of symptoms alone can expose contact-sport athletes to further injury before their recovery is complete. Task-related functional near-infrared spectroscopy (fNIRS) could be used to scan for abnormalities in the brain activation patterns of SRC athletes and help clinicians to manage their return-to-play. This study aims to show a proof of concept of mapping brain activation, using tomographic task-related fNIRS, as part of the clinical assessment of acute SRC patients. A high-density frequency-domain optical device was used to scan 2 SRC patients, within 72 h from injury, during the execution of 3 neurocognitive tests used in clinical practice. The optical data were resolved into a tomographic reconstruction of the brain functional activation pattern, using diffuse optical tomography. Moreover, brain activity was inferred using single-subject statistical analyses. The advantages and limitations of the introduction of this optical technique into the clinical assessment of acute SRC patients are discussed.

## 1. Introduction

Sport-related concussion (SRC) is a traumatic brain injury (TBI) characterized by a functional brain impairment in the absence of abnormalities on conventional neuroimaging (i.e., computerized tomography and magnetic resonance imaging (MRI)) [1,2].

In the United States, SRC is estimated to affect a range of between 1.6 and 3.8 million participants every year, with a continuous increase of athletes participating in contact sports [3,4]. These numbers could hide a more widespread burden as concussion can be underreported, underestimated, or denied by contact-sport players [5,6].

Following SRC, there is a timeframe, called the window of vulnerability, when a second impact could result in disproportional damage to the brain [7,8,9]. Avoiding impacts to the head in this window is important to SRC players as they are more prone to further SRC and musculoskeletal injuries than non-SRC players, possibly due to their inability to quickly process the evolution of the game and avoid bad collisions [10,11,12,13]. Therefore, the clinical decision regarding return-to-play is a pivotal moment in the management of contact-sport patients. 

Currently, the decision to return-to-play is guided by the resolution of symptoms related to SRC (e.g., headache, confusion) [1,14]. However, studies using neurocognitive testing (e.g., Immediate Post-Concussion Assessment and Cognitive Testing (ImPACT); balance tests) and neuroimaging techniques (e.g., magnetic resonance spectroscopy) questioned the resolution of the neurometabolic and functional impairments, which characterize SRC in symptom-free SRC players [15,16,17,18,19,20,21]. Thus, basing return-to-play decisions on symptoms alone may expose asymptomatic concussed players to a second impact before recovery is complete. In addition, McCrea et al. showed that a conservative approach, based on a symptom-free waiting period, would not necessarily result in a better outcome for players [13]. Symptom-rating scales can fail to give a complete picture of all the possible system impairments related to concussion (e.g., cognitive, vestibular, ocular-motor, hormonal), and even when these are combined with a neurological assessment (e.g., Sport Concussion Assessment Tool (SCAT)), this may result in an incomplete follow-up of all SRC components [22,23]. The limitations of clinical decisions based predominantly on symptoms highlight the need to merge clinical assessment of SRC patients with a multimodal neuromonitoring assessment to scan all the possible system impairments which can comprise SRC, in order that clinicians can manage return-to-play safely.

Task-related functional magnetic resonance imaging (fMRI) has been extensively used to map brain activation abnormalities in SRC to identify biomarkers for patient assessment [24]. However, fMRI is a complicated and expensive imaging technique which makes it challenging to apply on a large scale. The values reported by fMRI may also be inaccurate due to the programs used [25,26]. Finally, the modality of scanning could influence the brain activation pattern of players who are not familiar with the environment of the scanner and rules out the assessment of players who suffer from claustrophobia.

Task-related functional near-infrared spectroscopy (fNIRS) is a scalp-located neuroimaging technique that uses near-infrared light to measure changes in oxyhemoglobin (O_2_Hb) and deoxyhemoglobin (HHb). These changes are indicative of neurovascular coupling occurring with neuronal activation following a neurocognitive stimulus, similar to task-related fMRI [27,28]. Diffuse optical tomography (DOT) is an extension of fNIRS which, using models of light propagation within head structures and inversion approaches, tomographically maps the neurovascular response within the cortex with a resolution of about 1 cm [29,30,31]. Comparisons between cortical brain function monitored using task-related fNIRS and fMRI showed similar results [32,33,34,35]. So far, the use of task-related fNIRS in SRC has been limited to studies in chronic SRC athletes or after at least 2 weeks from injury [36,37,38,39,40,41,42]. Analyses on concussed athletes a few days from injury focused on cerebrovascular dysregulation during physical tasks (e.g., breath-holding task), limiting the optical analysis to the fluctuations of the levels of hemoglobin only, without reconstructing the signal into a tomographic image [43].

This study aims to illustrate a proof of concept of recording the brain activation pattern during 3 neurocognitive tests (i.e., Silent Word Generation (SWG), Digit Span (DS) backward, Symbol Search (SS)) in SRC players, within 72 h from injury, using optical tomographic reconstruction. The relatively short timeframe between injury and optical assessment highlights the possibility to perform optical neuromonitoring in acute SRC athletes as part of their early clinical assessments. The methods applied and the results obtained can pave the way for further fNIRS-DOT clinical studies during the assessment of acute SRC athletes.

## 2. Results

### 2.1. Co-Registration

**Figure 1:**
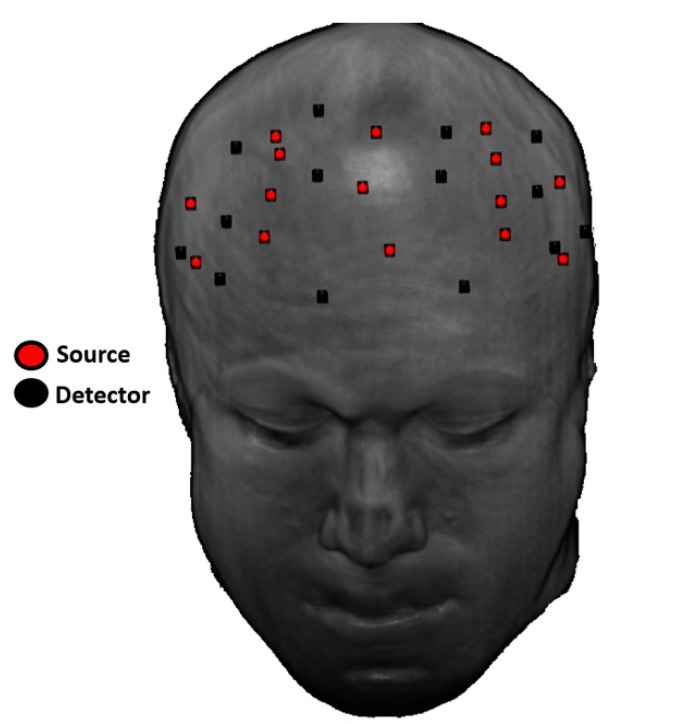
Illustration of the source-detector (SD) positions co-registered onto a structural magnetic resonance imaging (MRI) template.

The source-detector (SD) locations were co-registered on a structural MRI template, using the digitization of their positions and the subjects’ fiducials (Figure 1).

### 2.2. Oscillations of Oxyhemoglobin and Deoxyhemoglobin Levels

**Figure 2:**
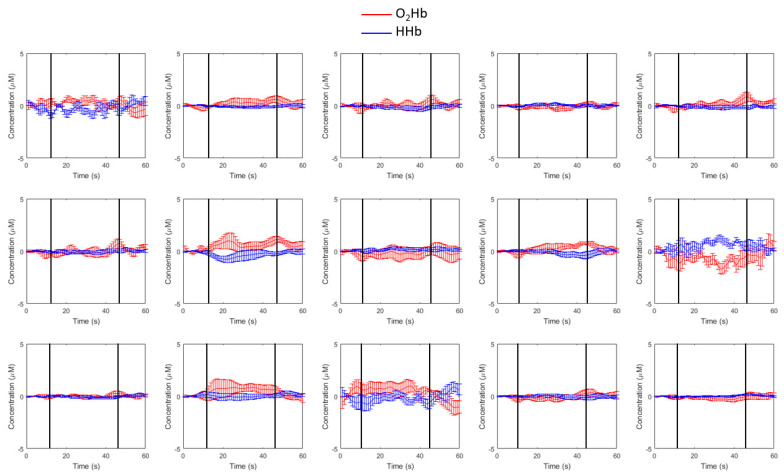
The 5 blocks average time-course response of changes in levels of oxyhemoglobin (O_2_Hb) and deoxyhemoglobin (HHb) in a single subject for the Silent Word Generation (SWG) task. The black vertical lines represent the start and the end of the task.

As evaluated by a task-related averaging approach at a channel level, during the SWG, changes in O_2_Hb and HHb levels were compatible with a neurovascular-mediated hemodynamic response. Due to brain activation, the functional hyperemia is expected to cause a few μM increase in the concentration of O_2_Hb, coupled with a smaller (2–3 times lower) decrease in HHb. The opposite pattern (decrease in O_2_Hb and increase in HHb) is often found in regions that are de-activated during the task. These oscillations happened at temporal resolutions typical of hemodynamic signals (few seconds). Examples of hemodynamic responses during the SWG are reported in Figure 2.

### 2.3. Statistical Models Used for Single-Subject Optical Tomographic Reconstruction of the Brain Activation Pattern

**Figure 3:**
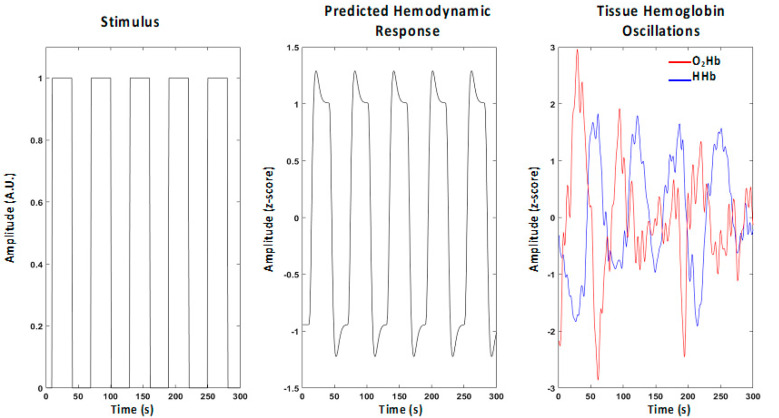
Scheme of application of the General Linear Model (GLM) in the analysis of the change of oxyhemoglobin (O_2_Hb) and deoxyhemoglobin (HHb) levels to detect the neuronal activation.

To spatially infer brain activity during SWG using the tomographic images, the General Linear Model (GLM) was used on O_2_Hb and HHb time-courses at a voxel-level [44,45,46]. For each voxel, the neuronal response was assumed to be a square wave locked to the stimulation, and this neuronal response was convolved with a canonical hemodynamic response function to obtain an expected pattern of hemoglobin oscillation due to brain activity. Finally, this response was correlated with the O_2_Hb and HHb time-courses in each voxel probed by the optical array to obtain a correlation coefficient and hence a statistical map (t-score map, Figure 3).

For the DS backward and SS tests, a different approach was utilized to infer brain activity based on the assumption of increased modulations in O_2_Hb and HHb during the task compared to the resting state. The absence of a defined alternation of stimulation periods and rest impaired the utilization of the GLM approach.

During brain activity, the standard deviation of O_2_Hb and HHb was evaluated in 10 s integration windows during the baseline period prior to and during the tasks. Using this approach, a statistical comparison was conducted between the populations of variabilities in O_2_Hb and HHb during the baseline period and the task. Hence, this procedure allowed us to infer brain activity without having a defined stimulation pattern, with the only limitation being that it was not possible to evaluate the sign of the oscillation in response to neural activity but only the amplitude.

**Figure 4:**
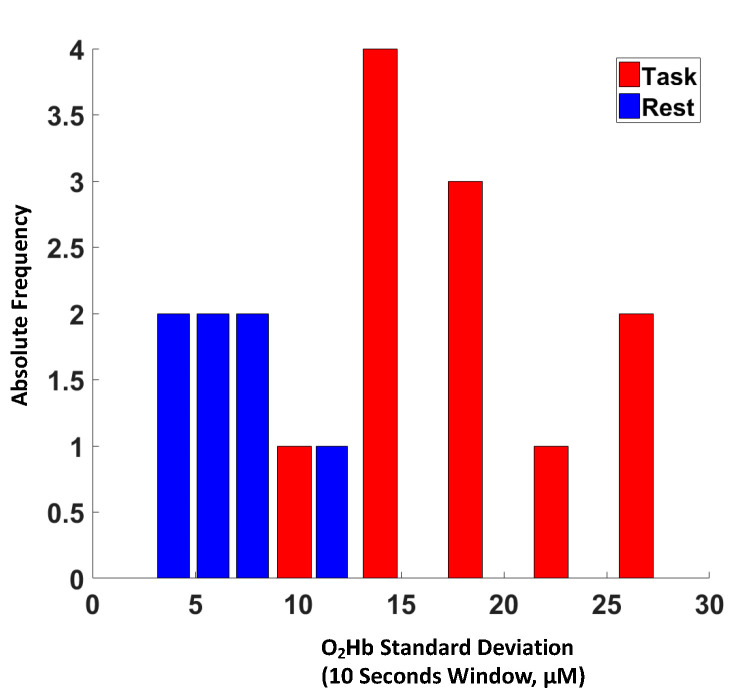
Example of histograms of standard deviations of oxyhemoglobin (O_2_Hb) for a voxel evaluated at a 10 s integration window during rest (blue bars) and the Symbol Search (SS) test (red bars). A clear increase in the average variability in O_2_Hb of a few μM is visible during the task, depicting plausible hemodynamic brain activity in the selected voxel.

Figure 4 shows an example of histograms of standard deviations of O2Hb for a particular voxel evaluated at a 10 s integration window during rest (blue bars) and the SS test (red bars). A clear increase in the average variability in O_2_Hb of a few μM is visible during the task, depicting plausible hemodynamic brain activity in the selected voxel. For each voxel, a statistical comparison (t-score) was performed evaluating the expected statistical difference (task vs. rest) between the average of the two populations reported in the figure.

### 2.4. Brain Activation Patterns Measured

**Figure 5:**
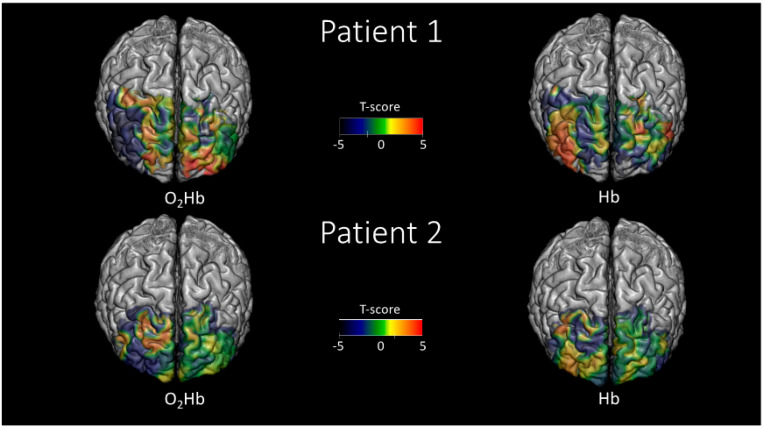
Statistical parametric maps (t-scores) evaluated employing the functional near-infrared spectroscopy-diffuse optical tomography (fNIRS-DOT) analysis and the General Linear Model (GLM) approach during the Silent Word Generation (SWG). The maps are reported in regions where the spatial sensitivity of the optical array was sufficient (up to an attenuation of the average optical sensitivity of 1000 times).

**Figure 6:**
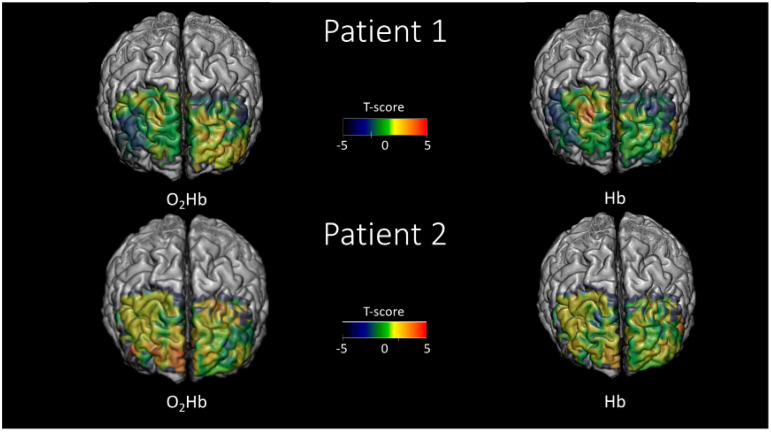
Statistical parametric maps (t-scores) evaluated employing the functional near-infrared spectroscopy-diffuse optical tomography (fNIRS-DOT) analysis and comparing the variability of the signal between task and rest for the Digit Span (DS) backward test. The maps are reported in regions where the spatial sensitivity of the optical array was sufficient (up to an attenuation of the average optical sensitivity of 1000 times).

**Figure 7:**
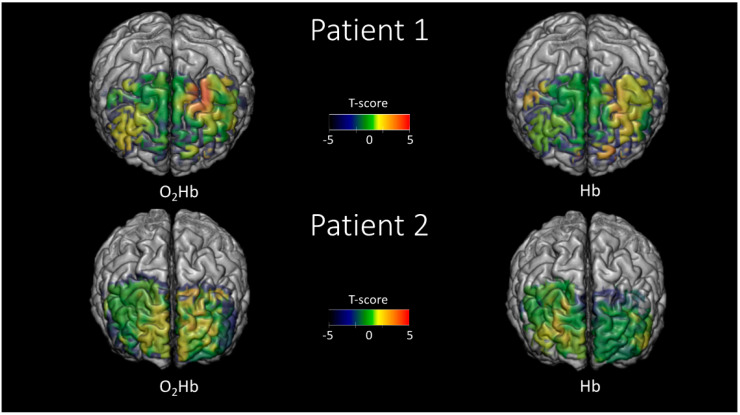
Statistical parametric maps (t-scores) evaluated employing the functional near-infrared spectroscopy-diffuse optical tomography (fNIRS-DOT) analysis and comparing the variability of the signal between task and rest for the Symbol Search (SS) test. The maps are reported in regions where the spatial sensitivity of the optical array was sufficient (up to an attenuation of the average optical sensitivity of 1000 times).

fNIRS-DOT statistical parametric maps for SWG, SD, and SS were performed for the two patients and the two hemoglobin forms (O_2_Hb and HHb). Moreover, they were reported in regions where the spatial sensitivity of the optical array was sufficient (up to an attenuation of the average optical sensitivity of 1000 times) (Figure 5, Figure 6 and Figure 7).

Figure 5 reports the statistical parametric maps (t-scores), obtained using the GLM approach, during the SWG. In both patients, a clear spatial anticorrelation in the maps of O_2_Hb and HHb is visible. The anticorrelation of O_2_Hb and HHb is highly indicative of the sensitivity to hemodynamic brain activity, which is the only physiological event that induces opposite oscillations of the two hemoglobin forms. It should be noticed that those t-scores above t = 1.65 or below t = −1.65 suggest a brain activation, as the null hypothesis probability (activation not significantly different from 0) within these values can be considered equal to the one with *p* < 0.05 in an uncorrected scenario.

Figure 6 reports the statistical parametric maps (t-scores), evaluated using the hemoglobin variability comparison between task and rest approach, during the DS backward test. In this case, O_2_Hb and HHb showed correlated maps, rather than anticorrelated ones as reported in Figure 5. This result is intrinsic to the nature of the analysis performed that evaluates only the statistical amplitude of the oscillations associated with brain activity, and not their sign.

Figure 7 reports the statistical parametric maps (t-scores), evaluated using the hemoglobin variability comparison between task and rest approach, during the SS test. In this case, for the same reason as described for Figure 6, O_2_Hb and HHb showed correlated maps. 

## 3. Discussion

### 3.1. Tomographic Task-Related Functional Near-Infrared Spectroscopy Assessment in Sport-Related Concussion within 72 Hours from Injury

These results show a proof of concept of application of task-related fNIRS-DOT in acute SRC patients while performing the SWG, DS backward, and SS tasks, during their clinical assessment. 

No changes were made to the neurocognitive tasks as presented in clinical practice. This is in agreement with other optical studies on patients (e.g., SRC, Alzheimer’s disease), which aimed to scan the brain activation patterns elicited during the clinical assessment (e.g., ImPACT) [37,47].

The 3 neurocognitive tasks used in this study had different modalities of execution: fixation on a screen in the SWG, repetition of numbers in the DS backward, and a pencil-paper task in the SS. This highlights the versatility of the optical recordings to different modalities of neurocognitive tasks. Different impairments related to SRC have been reported, which consequently demands the use of a battery of tests to scan SRC patients effectively, as a single test would not be sufficient to test the entire spectrum of possible dysfunctions [15,48]. The versatility of fNIRS-DOT to accommodate different modalities of execution of neurocognitive tests makes this neuroimaging technique particularly suitable for the clinical assessment of SRC patients. It should be noted that the execution of the SS or DS backward during fMRI scanning requires adaptation of the modalities of testing to suit the scanner or limitation to covert task responses [49,50]. It should also be highlighted that the SWG, unlike the other 2 tests, does not permit collection of behavior results. Limiting the analysis to the brain activation pattern alone can carry a bias in the analysis of the brain activation mapping, as this itself would be incorrectly assumed to be proof of the cognitive engagement, rather than seeking this proof in the behavior result [51].

The brain activation pattern had to be measured from the brain hemodynamic response using two statistical single-subject models: GLM and comparison in the variability of the signals between task and baseline for O_2_Hb and HHb. To extend the GLM to the optical data collected during DS backward and SS, the patterns of these tests must be edited into multiple blocks, similar to the template of the SWG. It should be noted that previous tomographic optical analysis on SRC patients used the GLM to map brain activation [37].

The optical analysis reported herein was recorded within 72 h of the patients’ injuries. The complex pathogenesis of concussion (e.g., energy crisis, altered neurotransmission) is reflected by different grades of severity of brain status according to the time of analysis since injury [52]. Behavior results in the neurocognitive tasks in SRC athletes can change with a progressive normalization of the parameters [53]. Therefore, optical analysis of the cortical activations related to these tests should be aimed at scanning patients within a short timeframe from injury so that early abnormalities can be detected. This would make a proper assessment of the severity of injury possible, and clinical decisions could be taken accordingly. Furthermore, the detection of early abnormalities can be useful to track their evolution during the patients’ recovery, and manage the follow-up accordingly.

### 3.2. Brain Activation Patterns Measured

#### 3.2.1. Silent Word Generation

The fNIRS-DOT results presented in this study showed stronger activation (positive t-scores for O_2_Hb coupled with negative HHb t-score) in the scanned areas of the prefrontal cortex in the left hemisphere (LH) than in the right hemisphere (RH). This is in agreement with the known brain language areas and fMRI results with SWG.

The language functions are mainly present in the LH [54,55]. The brain pattern is mainly compounded by two pathways: the dorsal pathway, which connects the Wernicke area, in the superior temporal gyrus, to the motor homunculus, in the anterior frontal gyrus; and the ventral pathway, which connects the Broca area, in the ventrolateral prefrontal cortex, to the middle and inferior temporal gyrus [56]. As expected by the activation pattern mentioned above, fMRI studies showed that the SWG is associated with brain activation of the prefrontal cortex, and stronger activation in the LH than in the RH [57,58,59]. 

#### 3.2.2. Digit Span

The fNIRS-DOT results showed higher variability in O_2_Hb levels during the DS backward test than the baseline in areas of the prefrontal cortex, which can be considered a sign of brain activation due to the task. 

These results are in agreement with previous NIRS studies which showed an increase in levels of O_2_Hb in the prefrontal cortex of both hemispheres during the DS backward and with studies using fMRI, transcranial magnetic stimulation, and positive emission tomography which suggested a crucial role of activation in the prefrontal cortex to perform the DS backward [50,60,61,62,63].

#### 3.2.3. Symbol Search

Similar to the DS backward results explained in Section 3.2.2, the fNIRS-DOT results showed an increased variance in O_2_Hb levels between the task and the baseline, which can be related to the neurocognitive activation triggered by the task.

To the best of our knowledge, this is the first example of tomographic reconstruction of brain activation related to SS as performed in clinical practice.

### 3.3. Design of Clinical Studies for Tomographic Functional Near-Infrared Spectroscopy in Sport-Related Concussion

Analysis of the behavior results from neurocognitive tests solely is currently insufficient for use for SRC clinical assessment [64,65,66]. Clinical studies on SRC patients could combine the brain activation patterns with the behavior results from the neurocognitive tests. This could give a better insight into the patents’ statuses and help clinicians to make decisions accordingly. 

The results from this study can pave the way for further clinical studies which aim to investigate abnormalities in the brain activation after SRC using task-related fNIRS-DOT, similar to previous task-related fMRI studies. The results herein presented can aid the design of the most suitable data collection (e.g., neurocognitive test) and analysis (e.g., statistical model) techniques for these future research studies.

It should also be mentioned that tomographic optical imaging (DOT) of the whole head can be used to map resting-state cortical activation, in a similar way to experiments done with resting-state fMRI in SRC [31,67].

### 3.4. Limitations

Several limitations should be mentioned about the study itself and the clinical application of task-related fNIRS-DOT in SRC. 

#### 3.4.1. Sample Size

The small sample size limited the analysis to single-subject statistical models, without extending to multiple-subject analysis [68]. Therefore, the capacity to retrieve the brain activation pattern across SRC athletes and to compare it between physiological and pathological states was not tested. Furthermore, the small sample size and the limitation to single-subject statistical analysis means that similarities in the brain activation patterns between subjects should be interpreted with caution.

#### 3.4.2. Confinement of the Results to the Illuminated Areas

The prefrontal cortex may not have been completely covered in the SD layout used. Due to the co-registration of the optical signal into an MRI template rather than a subject-specific structural MRI, the extension of the illuminated brain area in each patient could not be measured to a high accuracy. Therefore, conclusions on the prefrontal cortex activation are subject to the areas scanned.

#### 3.4.3. Limitations of the Statistical Model of the Variance of Levels of Oxyhemoglobin

Compared to the GLM, the analysis of variability of oscillations in O_2_Hb and HHb yields the disadvantage that the values obtained are agnostic to an increase or decrease of hemoglobin. Furthermore, task-related changes in the spontaneous hemodynamic oscillations may influence, completely or partially, the results obtained [27]. Consequently, the map plotted may not be related to the cortical activation only.

#### 3.4.4. Extracranial Tissue Interference 

Although the DOT approach intrinsically dampens the interference from the extracranial tissue (ECT), due to the positions of the probes on the scalp, hemodynamic interference may have partially affected the signal detected [69]. Moreover, the interference with the cerebral signal by the ECT hemodynamic may not be homogenous across the forehead [70].

The map of cortical activation during neurocognitive tests which require movement responses, such as the SS, may be less accurate than analysis during a task where participants stay still, such as the SWG, because movement artifacts and systemic motion-generated responses in the ECT could be additional confounding factors [27]. 

#### 3.4.5. Hemodynamic Impairments in Sport-Related Concussion

The task-related fNIRS-DOT analysis proposed in this study measures changes in the levels of O_2_Hb and HHb during the acquisition time, without assessing their baseline levels. Therefore, as proposed by Forcione et al., different levels of cerebral blood flow across the brain in SRC could influence the brain activation reported [24].

Furthermore, the lack of a gradual increase in difficulty of the neurocognitive tasks, as in the SWG or SS, and/or the inability to account for such an increase in the optical data analysis, as in the DS backward, exposes the brain activation pattern detected by fNIRS-DOT to confoundment by a pathological, non-linear, systemic hemodynamic response in SRC patients [24]. Future studies may address this problem by adding heart rate and/or blood pressure monitoring to the data acquisition method described herein.

#### 3.4.6. Multiple Patterns of Brain Activation 

There could be different brain activation patterns across individuals with different lifestyles. As a matter of fact, there are multiple variables, such as physical activity, education, type of sport practiced, which can affect the behavior results of neurocognitive tests and the cortical activation detected [71,72,73]. Therefore, non-standard brain activation would not necessarily link to impairments due to SRC but could instead be the result of comparisons between different group populations. Clinical studies should define markers in the brain activation pattern, which can be linked to dysfunctions related to SRC, by comparing brain activation patterns after SRC with the subject baseline values or with normative values from a non-concussed population, similar to the existing analysis in neurocognitive tests used in SRC [74].

## 4. Materials and Methods 

### 4.1. Patients

Two SRC rugby athletes (1 male, 35 and 23 years old) who were referred to the Birmingham concussion clinic at the Queen Elizabeth Hospital Birmingham or University of Birmingham (UK), right-handed, native English speakers, playing at amateur and professional levels, were scanned within 72 h from injury as part of the RECOS study (REpetitive COncussion in Sport) (IRAS ID: 216703; Ref. REC: 17/EE/0275) [75].

Patients took part in this study after providing informed written consent. The study conforms to the Declaration of Helsinki and it was approved by the East of England-Essex Research Ethics Committee on 22 September 2017.

### 4.2. Data Recording

#### 4.2.1. Optical Helmet

A custom-built optical helmet, as described by Tan et al., was used to position the optical probes on patients’ heads [76]. A custom-made MATLAB graphic user interface, NOMAD, Near-Infrared Optode Montage Automated Design (https://github.com/kylemath/nomad), developed by Mathewson et al., was used to evaluate the optimal SD locations on the helmet, in order to illuminate the prefrontal cortex of both hemispheres and avoid cross-talk between SD pairs in a time-multiplexing cycle of light-emission [77]. The prefrontal cortex was preferred over other areas because it was deemed to be activated by the neurocognitive tests used and due to the absence of hair on the forehead, which can impede the detection of optical signals.

#### 4.2.2. Digitization

A “3-space” FastTrak 3-D digitizer (Polhemus, Colchester, VT, USA) was used for digitizing the positions of the probes on the helmet and of three fiducial points (i.e., nasion, left and right pre-auricula). The positions of the pre-auriculae were defined using a T-bar, as described by Whalen et al. [78].

#### 4.2.3. Optical Device

15 detectors and 16 dual-wavelength sources of a frequency-domain near-infrared spectroscopy device (Imagent; ISS Inc., Champaign, IL, USA) were used to collect optical data. The location of one of the sources could not be resolved in the time-multiplexing cycle described in Section 4.2.1 and so it was not positioned on patients’ scalps. Each source emitted light at 830 nm and 690 nm wavelength, and they were modulated at 140 MHz. Optical data, accounting for a complete time-multiplexing cycle of all the sources, were sampled at 39.7365 Hz.

#### 4.2.4. Testing Set-Up

Patients were scanned while sat in a chair. A “double halo”, based on the description from Wheelock et al., was used to support the fibers [31]. Small plastic cones were affixed to the helmet’s holes, next to the probes, to secure them to the helmet and maintain good scalp-optode coupling during the recording. To maximize the scalp-optode coupling, care was taken to move the hair from the helmet’s holes before positioning the probes. 

**Figure 8:**
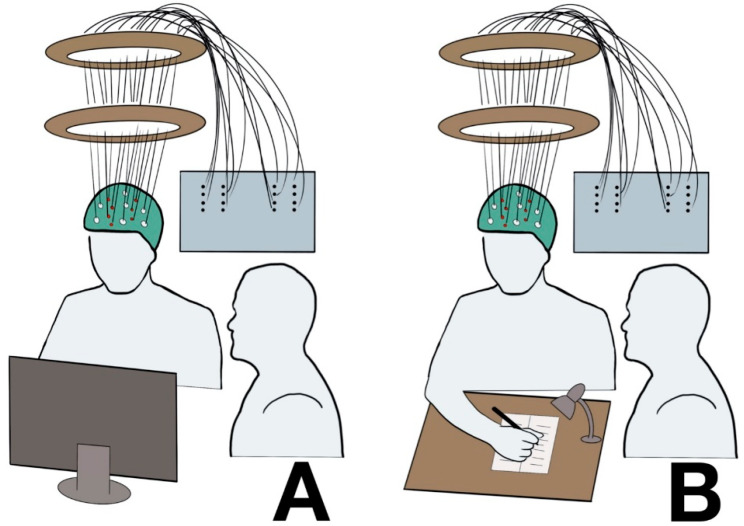
Representation of the test set-up. (**A**): Silent Word Generation (SWG), and Digit Span (DS) backward. (**B**): Symbol Search (SS).

An examiner sat close to the patients to interact with them if requested by the task (e.g., DS backward), and to control the correct execution of the tasks. A computer screen was placed in front of the patients (Figure 8A), and, during the SS, a small table was positioned in between to perform the task (Figure 8B). 

The light in the room was dimmed to reduce contamination by ambient light. During the SS, a small portable lamp was used to illuminate the table (Figure 8B). 

#### 4.2.5. Data Acquisition Protocol

**Figure 9:**
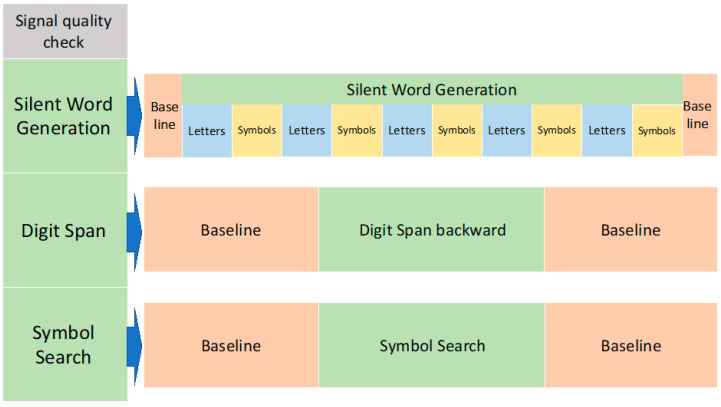
Experimental protocol.

Patients had to perform 3 neurocognitive tests commonly used in clinical practice: SWG, DS backward, and SS (Figure 9).

In order to investigate the feasibility of tomographic optical recordings during the tasks applied in clinical practice, no changes were made to the templates of the tests. Before the start of the recording, the tasks were explained, and patients had time to familiarize themselves with the tasks.

The optical data recordings were preceded by a signal quality check, and adjustments to the scalp-optode coupling were made as deemed necessary.

Each task was preceded and followed by 1 min of resting-state, during which patients had to stare at a cross projected on the screen. Two resting-state minutes in-between tasks were considered sufficient to allow hemoglobin levels to plateau and eliminate overlapping signals between tasks [79]. The 1 min of resting-state before the task was used as baseline in the statistical analysis of the variability of O_2_Hb and HHb, as explained in Section 4.3.

An examiner manually time-marked the different phases of the protocol. 

The duration of the protocol was approximately 30 min.

##### Silent Word Generation

The SWG is a common task used in clinical practice to assess the language areas before brain surgery [57].

The task was divided into 5 blocks. In each block, 3 slides showing a letter and 3 slides showing a nonsense symbol were projected on a screen each for 10 s, for a total block time of 60 s. The patients were instructed to think of as many English words as possible that began with the letter shown and to rest when the nonsense symbols were shown.

##### Digit Span Backward

The DS is part of the Wechsler Adult Intelligence Scales, Fourth Edition (WAIS-IV) test. The WAIS-IV has been used to evaluate different intellectual skills in TBI patients [80]. In particular, the DS backward is indicative of working memory and concentration skills [15]. 

The DS is compounded by a forward and backward version. In this study, only the backward version was carried out. The DS backward involved repeating a sequence of numbers in the reverse order to that in which they were announced. An examiner called a pair of sequences of 3 numbers each and, if the patient repeated them correctly, progressively longer sequences were given. When there was a mistake in both sequences, the task ended.

##### Symbol Search

Like the DS, the SS is part of the WAIS-IV, and it has been reported to be one of the most sensitive neurocognitive tests to TBI related impairments [80]. The SS is targeted at assessing visuospatial attention and cognitive processing speed [49].

Participants were presented with multiple lines of nonsense symbols. Each line comprised 2 sets of symbols, of length 2 and 5. Participants had to determine if a symbol appeared in both sets. If so, participants were instructed to strike through the repeated symbol, and if not, to strike through a “NO” box. Participants were instructed to complete as many lines as they could, to the highest possible accuracy, within 2 min. 

### 4.3. Data Analysis

Digitized optode’s locations were co-registered to a structural MRI template using a 3 fiducial-alignment approach [81]. The direct current (DC) intensity data (i.e., average measures of the amount of light produced by a specific source and reaching a particular detector during a multiplexed 1.6 ms interval) were normalized (by dividing them by their mean value) and converted into optical density (OD). Furthermore, the ODs were movement-corrected, and band-pass filtered between 0.01 Hz and 0.3 Hz (zero-lag, 4th order, Butterworth digital filter) [82]. For inspection of signal quality, a channel-based hemoglobin conversion was implemented employing the modified Beer-Lambert Law, with assumed hemoglobin extinction coefficients and tissue differential pathlength factors at the wavelengths of interest [83,84,85,86].

To generate a 3D reconstruction of changes in optical properties and hemoglobin oscillations in the frontal region investigated, a model of light propagation within the head (forward model) and an inverse procedure were implemented. The finite-element method (FEM) applied to the diffusion equation was used to estimate the forward model. The FEM software NIRFAST was used to model light propagation through the head and to compute the Jacobian (sensitivity) matrices of DC light intensity to absorption changes induced by hemoglobin oscillations in each SD couple [87,88]. “Fine” meshes (maximum tetrahedral volume 2mm^3^) were generated for FEM using the MATLAB software iso2mesh [89]. The heterogeneous head models were based on the segmentation of the MRI template. Segmentation of the skull and scalp, cerebrospinal fluid, white matter, and gray matter was performed using statistical parametric mapping functions [44]. Baseline optical properties (absorption coefficient [μa], reduced scattering coefficient [μs’] and refraction index [η]) of the tissues at the relevant wavelengths were taken from Tian et al. [90]. An inverse procedure based on energy minimization of the solution was used to convert intensity changes on individual channels to absorption changes in voxel space at each wavelength [88]. The modified Beer-Lambert law was inverted to evaluate O_2_Hb and HHb oscillations given absorption modulation at the 2 wavelengths employed. The extinction coefficients of the 2 forms of hemoglobin at the 690 nm and 830 nm wavelengths were extracted from Zijlstra et al. in similarity with the channel-based analysis [84].

Once tomographic reconstruction of O_2_Hb and HHb oscillations was obtained, 2 different statistical approaches were employed to evaluate brain activity. In particular, for the SWG test, the defined structure of the task permitted use of a GLM analysis [44]. In the GLM analysis, the nonsense symbol was considered as baseline, whereas the letters were considered as stimulation.

On the contrary, for the DS backward and SS, due to the absence of a defined pattern of stimulation, an ad-hoc analysis was implemented. In particular, an evaluation of the change in variability in O_2_Hb and HHb oscillations associated with brain activity was implemented by employing 10 s integration windows. The windows were non-overlapping, and they were evaluated in the 1 min of resting-state prior to beginning the task to estimate the baseline level and during the stimulation to estimate the task-induced brain hemodynamic modulation [91].

## 5. Conclusions

In conclusion, this study shows an application of task-related fNIRS-DOT in the analysis of brain activation in SRC patients within 72 h from injury. This study highlights the advantages and limitations of the use of this tomographic optical technique in the assessment of acute SRC patients. Future studies should aim to resolve or reduce the limitations met, identify biomarkers related to acute SRC in the optical assessment, and test the relevance of these biomarkers in the clinical decision process of return-to-play in SRC athletes.

## Abbreviations

SRC	Sport-related concussion
TBI	Traumatic brain injury
MRI	Magnetic resonance imaging
ImPACT	Immediate Post-Concussion Assessment and Cognitive Testing
SCAT	Sport Concussion Assessment Tool
fMRI	Functional magnetic resonance imaging
fNIRS	Functional near-infrared spectroscopy
O_2_Hb	Oxyhemoglobin
HHb	Deoxyhemoglobin
DOT	Diffuse optical tomography
SWG	Silent Word Generation
DS	Digit Span
SS	Symbol Search
SD	Source-detector
μM	Micromolar
GLM	General Linear Model
LH	Left hemisphere
RH	Right hemisphere
ECT	Extracranial tissue
WAIS-IV	Wechsler Adult Intelligence Scales, Fourth Edition
DC	Direct current
OD	Optical density
FEM	Finite-element method
